# Decadal stability in coral cover could mask hidden changes on reefs in the East Asian Seas

**DOI:** 10.1038/s42003-023-05000-z

**Published:** 2023-06-10

**Authors:** Y. K. S. Chan, Y. A. Affendi, P. O. Ang, M. V. Baria-Rodriguez, C. A. Chen, A. P. Y. Chui, Giyanto, M. Glue, H. Huang, C-Y. Kuo, S. W. Kim, V. Y. Y. Lam, D. J. W. Lane, J. S. Lian, S. M. N. N. Lin, Z. Lunn, C. L. Nañola, V. L. Nguyen, H. S. Park, Suharsono, M. Sutthacheep, S. T. Vo, O. Vibol, Z. Waheed, H. Yamano, T. Yeemin, E. Yong, T. Kimura, K. Tun, L. M. Chou, D. Huang

**Affiliations:** 1 Department of Biological Sciences, National University of Singapore, Singapore, Singapore; 2 Institute of Ocean and Earth Sciences, Universiti Malaya, Kuala Lumpur, Malaysia; 3 Institute of Space and Earth Information Science, The Chinese University of Hong Kong, Shatin Hong Kong SAR, China; 4 Marine Science Institute, University of the Philippines Diliman, Quezon, Philippines; 5 Biodiversity Research Centre, Academia Sinica, Taipei, Taiwan; 6 Research Center for Oceanography, National Research and Innovation Agency (BRIN), Jakarta, Indonesia; 7 Fauna & Flora International, Phnom Penh, Cambodia; 8 South China Sea Institute of Oceanology, Chinese Academy of Sciences, Guangzhou, China; 9 School of Biological Sciences, The University of Queensland, Brisbane, Australia; 10 Global Coral Reef Monitoring Network, International Union for the Conservation of Nature, Washington D.C., USA; 11 Marine Spatial Ecology Lab, School of Biological Sciences, The University of Queensland, Brisbane, Australia; 12 Lee Kong Chian Natural History Museum, National University of Singapore, Singapore, Singapore; 13 Universiti Brunei Darussalam, Bandar Seri Begawan, Brunei Darussalam; 14 Fauna & Flora International, Yangon, Myanmar; 15 University of the Philippines Mindanao, Davao, Philippines; 16 Institute of Oceanography, Vietnam Academy of Science and Technology, Nha Trang, Vietnam; 17 Korean Institute of Ocean Science and Technology, Seoul, South Korea; 18 Department of Biological Sciences, Ramkhamhaeng University, Bangkok, Thailand; 19 Department of Fisheries Conservation, Ministry of Agriculture, Phnom Penh, Cambodia; 20 Borneo Marine Research Institute, Universiti Malaysia Sabah, Kota Kinabalu, Malaysia; 21 National Institute for Environmental Studies, Tsukaba, Japan; 22 Faculty of Science, Ramkhamhaeng University, Bangkok, Thailand; 23 Reef Check Brunei, Bandar Seri Begawan, Brunei Darussalam; 24 Global Coral Reef Monitoring Network East Asia Region, Tokyo, Japan; 25 Palau International Coral Reef Center, Koror, Palau; 26 National Biodiversity Centre, National Parks Board, Singapore, Singapore; 27 Tropical Marine Science Institute, National University of Singapore, Singapore, Singapore; 28 Centre for Nature-based Climate Solutions, National University of Singapore, Singapore, Singapore

**Keywords:** Community ecology, Tropical ecology

## Abstract

Coral reefs in the Central Indo-Pacific region comprise some of the most diverse and yet threatened marine habitats. While reef monitoring has grown throughout the region in recent years, studies of coral reef benthic cover remain limited in spatial and temporal scales. Here, we analysed 24,365 reef surveys performed over 37 years at 1972 sites throughout East Asia by the Global Coral Reef Monitoring Network using Bayesian approaches. Our results show that overall coral cover at surveyed reefs has not declined as suggested in previous studies and compared to reef regions like the Caribbean. Concurrently, macroalgal cover has not increased, with no indications of phase shifts from coral to macroalgal dominance on reefs. Yet, models incorporating socio-economic and environmental variables reveal negative associations of coral cover with coastal urbanisation and sea surface temperature. The diversity of reef assemblages may have mitigated cover declines thus far, but climate change could threaten reef resilience. We recommend prioritisation of regionally coordinated, locally collaborative long-term studies for better contextualisation of monitoring data and analyses, which are essential for achieving reef conservation goals.

## Abstract (web summary)

An overview of coral reefs across East and Southeast Asia spanning >24,000 surveys in almost 2,000 sites finds that coral and macroalgal cover are surprisingly stable through time, though correlated environmental variables reveal hidden impact.

## Introduction

Coral reefs are some of the most diverse ecosystems on Earth, supporting numerous species, ecosystem services and livelihoods^1–3^. Against the backdrop of global sea-level rise, ocean warming and acidification, coral reefs across the world are facing unprecedented degradation^4–6^. Local anthropogenic stressors, including coastal urbanisation, overfishing and pollution vary among regions but can interact with global stressors to exacerbate reef degradation^7–11^. Identifying the changes that have affected reefs over longer time frames necessitates the analysis of long-term datasets. While long-term monitoring data enable changes that affect the health of the ecosystems to be detected, allowing for early intervention by influencing management and policy decisions^12–14^, the availability of data remains limited for many reefs^15,16^. Recent monitoring efforts have improved the spatial scale and precision of their products with technological advancements and dedicated resources^14,17,18^, but past monitoring data are still critical for establishing appropriate baselines to compare ecosystems across larger temporal windows^19,20^. Indeed, a crucial objective of long-term ecological research is to extend the analysis of reef trends back to the earliest baseline against which more contemporary changes can be compared and interpreted^21,22^.

Various long-term coral reef monitoring programs have been established for many reef regions^14,17^. In the Great Barrier Reef, reef monitoring is conducted as part of the Great Barrier Reef Marine Monitoring Program^23^, while various programs exist for the Caribbean such as the Caribbean Coastal Marine Productivity^24^. Within the Great Barrier Reef, long-term monitoring revealed high recent declines and differing trajectories of coral and macroalgal cover on reefs across different continental shelf zones^25,26^, with a recent report reporting dramatic coral recovery^27^. The Caribbean monitoring programs produced clear evidence for the region-wide phase shift from coral to macroalgal cover, later revealed to have resulted from a myriad of interplaying factors including parrotfish and sea urchin declines^28–30^. In comparison, long-term monitoring studies are more limited and have not been as coordinated among the reefs in the Northeast and Southeast Asia regions, typically with Reef Check initiatives, national or non-governmental organisations and academic institutions running distinct programs^16^. This is in spite of the area containing some of the world’s largest and most diverse reef areas nestled among archipelagic states with long coastlines^31,32^, encompassing most of the Coral Triangle^33^, as well as representing substantial coral cover^31,34^ and high diversity of corals and other reef organisms^35–38^. Incidentally, this high diversity also makes it challenging to assess resilience as different species can fill the same niche at different reefs, so comparisons across the region would be difficult^39–41^. As Indo-Pacific reefs continue to face serious threats from both global and local stressors^42,43^, establishing a historical ecological baseline across the East Asian Seas is more critical than ever to understand the spatial distribution and environmental correlates of the wide-ranging changes on these reefs over the decades they have been monitored^44,45^.

**Fig. 1:**
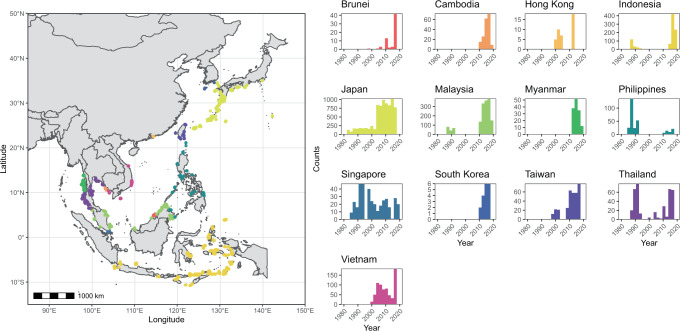
Map of the East Asian Seas region. Survey sites across 13 localities (left) and histogram of reef survey counts through time for the regional dataset (right).

In the absence of coordinated long-term monitoring programs in the region, we leveraged data gathered by the Global Coral Reef Monitoring Network East Asia (GCRMN EA) node. The network was established through the regional partnership among national coordinators representing the Northeast Asia (China, Hong Kong, Taiwan, Japan, and South Korea) and Southeast Asia nodes (Brunei Darussalam, Cambodia, Indonesia, Malaysia, Myanmar, Philippines, Singapore, Thailand, and Vietnam) of the GCRMN (Fig. 1). The present study thus seeks to consolidate and model available benthic data to test for spatial and temporal variations in the benthic cover of hard corals and macroalgae throughout the East Asia region, providing a baseline for further investigations into more acute reef trends. These two specific benthic parameters were chosen because they are universal and important for benthic characterisation. They are also some of the easiest to measure and compare across a large region. While some survey methods record coral growth forms and sometimes in greater taxonomic resolution, these data were not consistent and available across the region through time to be analysed precisely. This study builds on past qualitative and disparate trend reports such as Kimura et al.^46^ as well as recent quantitative reports for other regions including the Western Indian Ocean and the Pacific islands^30,34,47,48^.

Specifically, we collate, synthesise, and analyse benthic datasets from within the GCRMN EA nodes to present a rigorous, data-driven trend of the region’s coral reefs from the 1980s through 2019. We hypothesise that coral cover has generally declined over time, with reefs experiencing recovery following disturbance events, while macroalgae have increased in abundance on the reefs in the region. We also hypothesise that changes in benthic cover are associated with both anthropogenic pressures and sea surface temperature rise, with both factors negatively correlated with coral cover and positively correlated with macroalgal cover. To test these hypotheses, this study identifies variations in hard coral and macroalgal cover among localities over nearly four decades, tests for associations between various environmental and socio-economic factors with reef benthic cover, and contextualises the implications of such changes for regional reef management and monitoring into the future.

## Results

### Coral and macroalgal cover through time

We compiled data from a total of 24,365 surveys at 1,972 reef sites throughout the East Asia region surveyed from 1983 to 2019 (Fig. 1; Supplementary Table 1). These data were collected via line- and point-intercept transects, timed swims and photo quadrats, with one to six replicates per survey. While the dataset spans 37 years of surveys, benthic data from the first ~15 years were relatively limited, with only Japan and Singapore having consistent data throughout the 1980s to 2000s while other localities had sporadic data at much fewer time points (Fig. 1). Data coverage increased with time, especially after the 2000s, but this was neither consistent throughout the different localities nor commensurate with their reef areas. Japan had the most temporally consistent coverage of coral data throughout the survey periods, with Singapore having the next most consistent data through time. Malaysia, Indonesia and Vietnam showed substantial increases in data availability through time, with most of these data representing the post-2000s. Apart from the lack of macroalgal data for Japan’s datasets and a small number of other localities, general data availability patterns through time followed the coral data (Supplementary Table 1).

**Fig. 2:**
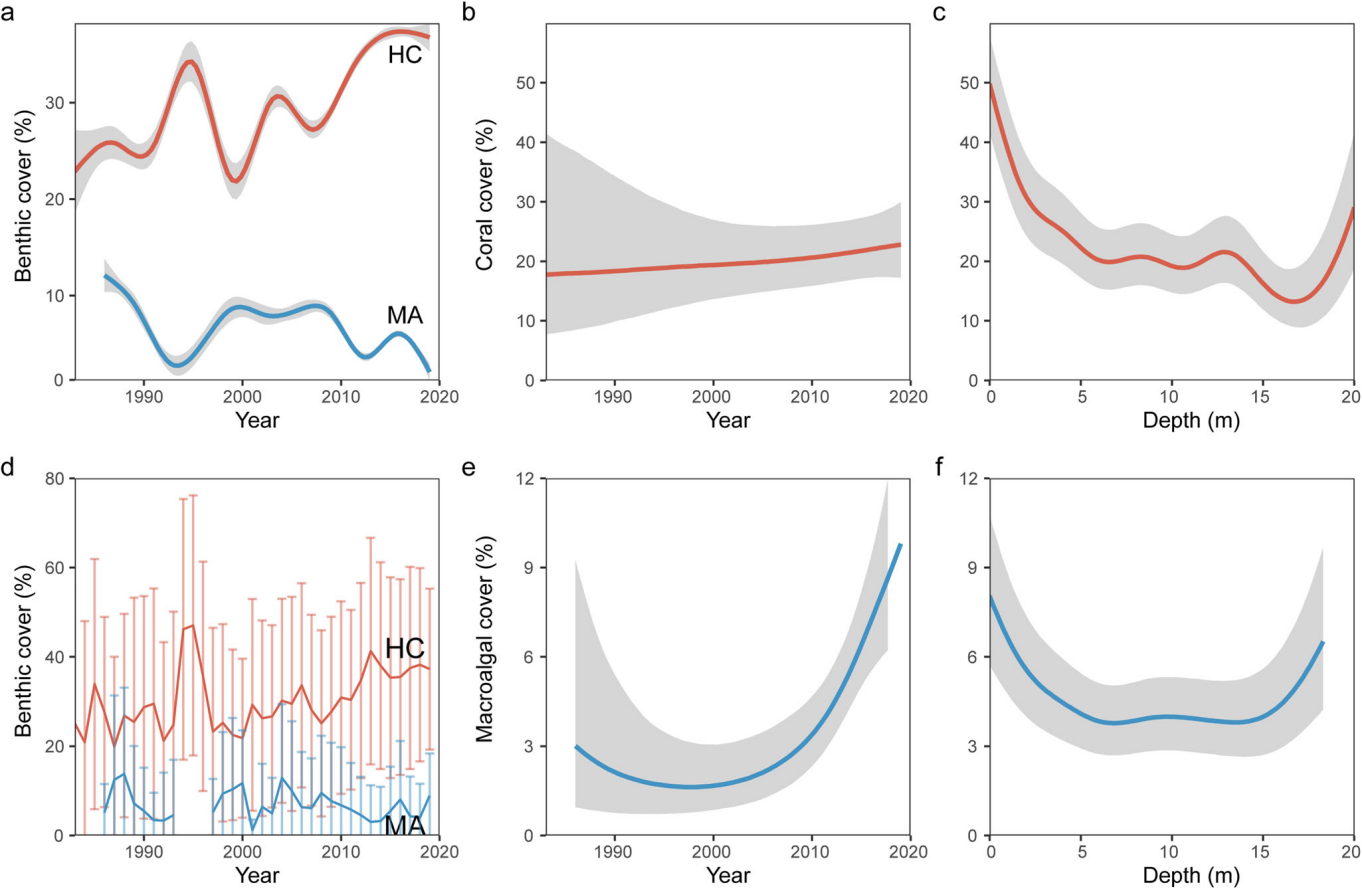
Coral (HC) and macroalgal (MA) cover changes over time and depth. **a** LOESS coral (red) and macroalgal (blue) cover means across time. **b** Modelled Bayesian GAMM coral cover with time. **c** Modelled Bayesian GAMM coral cover with depth. **d** Coral (red) and macroalgal (blue) cover annual means and standard deviations across time. **e** Modelled Bayesian GAMM macroalgal cover with time. **f** Modelled Bayesian macroalgal cover with depth. Shaded regions represent the modelled credible intervals.

Overall, temporal trends in coral and macroalgal cover exhibited stable patterns over the study period (Fig. 2). Specifically, the Locally Estimated Scatterplot Smoothing (LOESS) plots concur with the locality smoothers produced by the Bayesian hierarchical model, both of which indicated a mean coral cover over time moving slightly upwards through most of the study period (Fig. 2a, b). Both results also highlighted that cover had only slight variation centred around 25% (Fig. 2a) and 20% (Fig. 2b) respectively. While there appeared to be a slight increase in coral cover over time, credible intervals in the modelled trends (Fig. 2b) were relatively large before the 2000s due to the limited data available, with fewer surveys conducted and fewer localities represented. Coral cover declined with increasing depth, sharply decreasing from 48% at 0 m to a stable 20% at 6 m before decreasing again past 13 m to 14% (Fig. 2c). Coral cover increased slightly past 17 m to about 25% with some of the variation explained by common depths in surveys (Supplementary Table 1). Consistent with the coral cover trends, macroalgal cover for both plots ranged between 0% and 10% throughout the study period (Fig. 2d, e). The modelled trend showed a clearer increase over time compared to the LOESS plot, with macroalgal cover increasing from 3% before 2000 to 6% in 2019 (Fig. 2e). Across depths, macroalgal cover declined from 8% at the surface to 4% between 5 m and 15 m before increasing to 7% at >15 m depth (Fig. 2f).

**Fig. 3:**
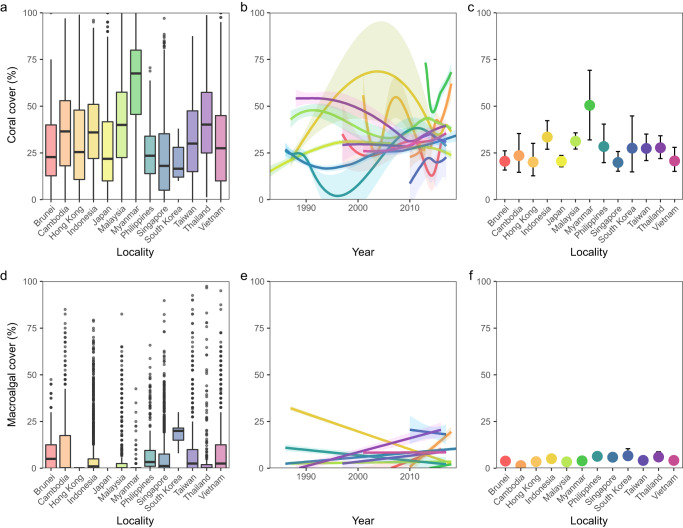
Coral and macroalgal cover variations among reef localities. **a** Boxplot of locality-level coral cover means. **b** LOESS coral cover means by locality through time. **c** Modelled Bayesian GAMM locality-level coefficients of coral cover. **d** Boxplot of locality-level macroalgal cover means. **e** GAM macroalgal cover means by locality through time. **f** Modelled Bayesian GAMM locality-level coefficients of macroalgal cover. Shaded regions represent the modelled credible intervals.

There were considerable variations in coral cover and how these changed temporally among the localities examined according to the data visualisations and Bayesian-modelled coefficients (Fig. 3a–c, Supplementary Table 2, Supplementary Fig. 1). Across all localities, coral cover varied widely at individual survey sites ranging from 0% to 100% with means generally within 20–40% barring Myanmar with higher cover (Fig. 3a). All localities generally showed fluctuating patterns of cover change (Fig. 3b). Coral cover based on modelled coefficients for several localities including Brunei, Japan, Singapore, and Vietnam were low on average at 20–25% and had relatively small variability. Other localities such as Cambodia and Hong Kong had similarly low coral cover of 20–25% but higher variability, whereas Indonesia, Malaysia, Philippines, South Korea, Taiwan, and Thailand had slightly higher coral cover of ~30% and high variability. Among all localities, Myanmar stood out with even higher coral cover and large standard deviation. Myanmar and South Korea showed higher variability in coral cover compared to the other localities, likely due to data scarcity. Locality-level modelled trend lines also showed generally consistent coral cover through time with either slight increases or decreases from 12–25% to ~25% in the present. For example, there was a gradual decline of mean cover from 50% to 30% in Thailand up until 2010 before recovery, while most other localities like Singapore, Taiwan and Vietnam showed little change over their respective survey periods (Supplementary Table 2). The large variability was also seen in the unadjusted conditional Bayesian *R*^2^ (mean ± standard error; 0.532 ± 0.003) being much higher than the unadjusted marginal Bayesian *R*^2^ value (0.155 ± 0.020), highlighting that most of the variation was explained by the random effect variables site and location instead.

Temporal trends in macroalgal cover also differed between localities, though variations were less clear than for coral cover (Fig. 3d–f, Supplementary Table 3, Supplementary Fig. 2). Across all localities, macroalgal cover had much smaller quartiles between 0% and 10% with South Korea having slightly higher macroalgal cover (Fig. 3d), and trends over time were less variable than for coral cover (Fig. 3e). In general, modelled coefficients of macroalgal cover were low across all regions, with the highest mean macroalgal cover found in Indonesia, Philippines, Singapore, South Korea, and Thailand, being only 5–7%. Even lower macroalgal cover was found in the remaining localities, with Brunei, Hong Kong, Malaysia, Myanmar, Taiwan, and Vietnam showing mean cover of 3–4%, and Cambodia with the lowest mean cover of slightly above 1%. Bayesian-modelled macroalgal cover at each locality was more stable over time compared to the coral cover trends, mostly ranging between 0% and 10% throughout the survey period, except Indonesia where macroalgal cover declined from 20% to 2% and Cambodia where it increased post-2010 with an especially large credible interval before 1990 (Supplementary Fig. 2). Bayesian *R*^2^ of the macroalgal model was much lower with an unadjusted Bayesian conditional *R*^2^ (0.295 ± 0.010) and an unadjusted Bayesian marginal *R*^2^ (0.071 ± 0.018), with most of the variation explained by the random effect variables site and location.

### Effects of coral bleaching events on coral cover

**Fig. 4:**
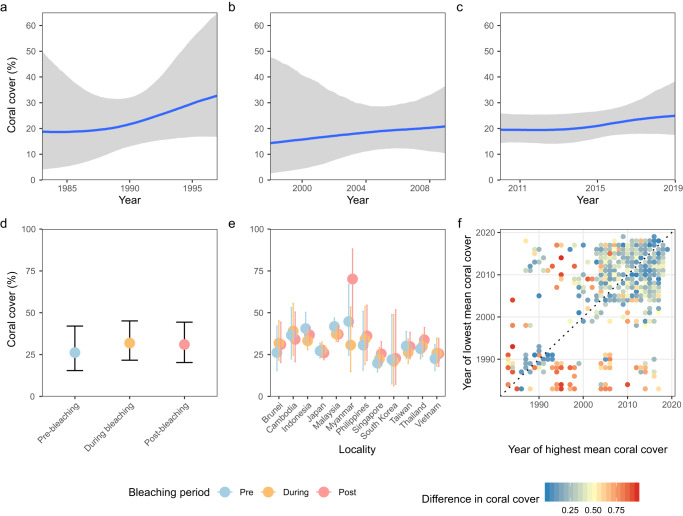
Coral cover through major coral bleaching events in the East Asia region. Data subsets individually modelled for each time period to determine if main results were sensitive to the time periods separated by major coral bleaching events (**a**) before the first major bleaching events of 1998 **b** between major bleaching events from 1998 to 2009 and **c** from 2010. **d** Modelled coral cover between bleaching periods showing lower coral cover before and after bleaching events (±2 years) than during bleaching events. **e** Modelled coral cover across bleaching periods for each locality showing large variations among pre-, during and post-bleaching cover. **f** Sites plotted by years of maximum and minimum cover to show the differences in coral cover between the two time points. Points above the line indicate greater recency of low cover compared to high cover in proportion to the distance from the line with colours representing the degree of difference in cover. Data show most points were blue and near the line, indicating little change in cover throughout most of the study period, while few red points were farther from the line, indicating that only few sites showed drastic changes over the study period. Shaded regions and error bars represent the modelled credible intervals.

To validate the inferred temporal trends and determine the degree to which the global-scale coral bleaching events affect coral cover, we organised the dataset into three roughly decadal time periods separated by the global-scale coral bleaching events in 1998 and 2010. When these data subsets were modelled using the same Bayesian GAMM model structure as above, the analysis indicated similar trends among the three time periods (Fig. 4a–c), with only a difference between the end of the first pre-bleaching survey period (Fig. 4a) and the beginning of second survey period around 1998 (Fig. 4b). This discrepancy was indicative of some effect of the 1998 global-scale coral bleaching event on coral cover that was not consistent among the localities. Since Myanmar and Japan had the highest coral cover and the most consistent temporal trends respectively, we tested if they may have had a disproportionate influence on the results by rerunning the models without these two localities. The overall trends were not discernibly different with the reduced data subsets (Supplementary Fig. 3).

Three global-scale coral bleaching events occurred within the timeframe of the surveys analysed here—in 1998, 2010 and 2016. To determine if there were region-wide bleaching effects on coral cover, we further subset the data to within 2 years of the bleaching events and assigned the surveys to either pre-bleaching, during bleaching, and post-bleaching with reference to the coral bleaching events. We then tested if these individual bleaching events and the timing of events relative to the surveys affected coral cover using a Bayesian hierarchical model with an interaction between the locality and bleaching event. The simplified model showed that coral cover was the lowest pre-bleaching, with similar cover during bleaching and post-bleaching (Fig. 4d). The interaction between locality and bleaching periods was variable, with some localities showing the same pattern as the general model likely indicating potential effects of past bleaching events (Brunei, Cambodia, Japan and Vietnam) (Fig. 4e). Some localities showed the typical decline during bleaching, followed by recovery (Indonesia, Malaysia, Myanmar, South Korea and Taiwan), and others showed more resilience with cover increasing from pre-bleaching to during to post-bleaching (Philippines, Singapore and Thailand) (Fig. 4e). When comparing temporal patterns of coral cover for each individual reef and the respective year surveyed, no clear patterns were observed (Fig. 4f). The biggest changes for individual reefs were spread across years, with no particular time periods exhibiting disproportionate declines, highlighting that reefs throughout the region likely had differentiated declines and recovery through time (Fig. 4f). Lag effects were not recovered in the model; variation was parsed mostly through the temperature variables, with similar smoothers seen across the models for different years and lag times (Supplementary Fig. 4).

### Environmental and socio-economic correlates of reef benthic cover

To test the effects of environmental and socio-economic variables on benthic cover, we analysed these variables and benthic cover proportions via multi-modal inference with model selection and aggregation performed through an information criterion approach. In most cases, both coral and macroalgal cover showed inverse correlations with temperature (Sea Surface Temperature (SST), SST Anomalies (SSTA), Degree Heating Weeks (DHW) and Bleaching Alert Levels (BAA)), nutrients (Net Primary Productivity (NPP)) and anthropogenic (population and distance) variables. Variables associated with coral cover were aggregated using only two models with similar variables (Table 1), while macroalgal associations were aggregated from more models with considerably different sets of variables (Table 2). Nevertheless, most of the variables were shown to have significant correlation with coral and macroalgal cover.

**Table 1:** Correlated variables within multi-model averages showing category of variables, number of models they appear in, their effects on coral cover with their estimates, standard error, credible intervals and significance at α = 0.05 (*: significant, #: variable significant but not for all levels within variable).

Variables in coral model (No. of inclusions / total no. of variables)		Effect	Estimate	Adj SE	2.5% CI	97.5% CI	p-value
**Temperature**							
baa_max	1/2	−	−0.00592	0.00138	−0.00864	−0.00321	1.87e-05*
**Nutrient**							
npp_max	2/2	+	0.0852	0.0142	0.0574	0.113	<2e-16*
npp_sd	2/2	−	−0.0799	0.0142	−0.108	−0.0521	<2e-16*
**Anthropogenic**							
Locality	2/2	±					#
pop_est_100km	2/2	−	−0.0392	0.00634	−0.0517	−0.0268	<2e-16*
**Miscellaneous**							
Depth	2/2	−	−0.00847	0.000708	−0.00986	−0.00708	<2e-16*
reef_area_100km	1/2	−	−0.0316	0.00821	−0.0477	−0.0155	0.000118*

Please bold “Temperature”, “Nutrient”, “Anthropogenic” and “Miscellaneous” section headers within the table.

**Table 2:** Correlated variables within multi-model averages showing category of variables, number of models they appear in, their effects on macroalgal cover with their estimates, standard error, credible intervals and significance at *α* = 0.05. (*: significant, #: variable significant but not for all levels within variable).

Variables in macroalgal model (No. of inclusions / total no. of models)		Effect	Estimate	Adj SE	2.5% CI	97.5% CI	*p*-value
**Temperature**							
dhw_max	3/6	+	0.00282	0.000944	0.000969	0.00467	0.002823*
sst_max	5/6	−	−0.0158	0.00364	−0.0230	−0.00870	1.39e-05*
sst_mean	4/6	+	0.0129	0.00352	0.00604	0.0198	0.000236*
sst_min	1/6	+	0.00647	0.00222	0.00211	0.0108	0.003594*
ssta_max	1/6	−	−0.00416	0.000951	−0.00603	−0.00230	1.19e-05*
**Nutrient**							
npp_max	1/6	+	0.00869	0.00356	0.00171	0.0157	0.014679*
npp_sd	1/6	+	0.00656	0.00370	−0.000693	0.0138	0.076267
**Anthropogenic**							
Locality	6/6	±					#
pop_est_100km	6/6	+	0.0269	0.00480	0.0175	0.0363	<2e-16*
**Miscellaneous**							
Depth	6/6	−	−0.00255	0.000396	−0.00333	−0.00177	<2e-16*
Year	1/6	+	0.000491	0.000245	0.0000107	0.000972	0.045118*

*Significant. #: variable significant but not for all levels within variable.

Coral cover was negatively affected by some environmental variables like max BAA and NPP SDs, while max NPP showed a positive correlation. Anthropogenic impacts were apparent based on the negative correlation between human population and coral cover. Additionally, while the negative correlation between depth and coral cover was expected due to attenuating light with depth, the negative correlation between reef area and cover was surprising. While locality showed up as a variable within the aggregated model, no specific localities were significant except for Hong Kong, indicating that locality was not a strong predictor of coral cover (Supplementary Table 4). For macroalgal cover, six models were aggregated, with temperature variables involved frequently in various forms. Temperature variables DHW max, SST mean, SST min were positively correlated, while SSTA max and SST max were negatively correlated, with macroalgal cover. NPP max and population size also had positive correlations with macroalgal cover. Year and depth were in the aggregated model with positive and negative correlations respectively, but few localities—Hong Kong, Malaysia and Singapore—were significant despite locality being selected as well (Supplementary Table 5).

## Discussion

The generally stable coral cover through time of reefs in the East Asian Seas is inconsistent with previous studies that show region-wide declines over the last 30 years^49,50^. The regional mean coral cover estimated here appears stable at ~25%, which is consistent with data from previous analyses of Indo-Pacific reefs post-1980s^49^, and similar to other regional means such as in the Great Barrier Reef^23^ and Caribbean^29^ before declines in the 2010s and 1980s respectively. We also did not find evidence of geographic variability in coral cover declines. In line with how coral cover has remained stable, macroalgal cover throughout the East Asian Seas region has also remained relatively consistent with only minor increases detected. The modelled results showing macroalgal cover increase from 3% to 6% can become concerning if these increases continue into the future, but current levels remain relatively low compared to other regional studies^30,51^. Importantly, low macroalgal cover may also potentially affect coral growth, such as through competitive exclusion and other negative interactions, so continued monitoring of both coral and macroalgal cover is necessary for assessing reef condition^28,52^.

The low and generally stable macroalgal cover seen in East Asia contrasts with the sharp increase in macroalgal cover in the Caribbean following the decline of *Diadema* urchin populations^28,30^, and highlights differences in reef trajectories despite apparent coral losses following major bleaching events. Indeed, unlike in the Caribbean reefs where macroalgal phase shifts have occurred following the herbivore declines^30^, there is no evidence here pointing to clear coral cover loss associated with macroalgal blooms in East Asian reefs. However, it is also important to note that some reefs in the Indo-Pacific have been shown to undergo phase shifts towards dominance of other benthic organisms, such as sea anemones, soft corals and zoanthids, which were not quantified in our study^51,53,54^. For example, Reimer et al.^55^ reported alternative phase shifts among reefs in Malaysia and Japan that were eventually dominated by zoanthids.

Despite efforts to amass extensive data throughout the region and across time, the paucity of data prior to the 1990s contributed to the large credible intervals in the model outcomes for this period that have obfuscated levels of coral cover during the early years of reef monitoring. Certainly, changes in coral cover prior to 1980 could not be inferred with the current data. Anecdotal evidence and historical accounts, however, do suggest that there were past declines from reef states with higher coral cover. For instance, more than half of the reefs surveyed in Malaysia and Thailand before the 1990s exhibited much higher coral cover (>50%)^21,50,56^. On the one hand, it is possible that high coral cover reefs were more common in the past and these reefs may have experienced declines in the years prior to modern monitoring surveys, as inferred by previous large-scale analyses^49^ and expert opinion^50^. On the other hand, improved management of reefs and conservation interventions in the region, especially since the mid-1990s, may have reduced the decline in certain reefs^42,44,57^. Verifying scales of historical losses and ascertaining how common pristine high coral cover reefs were remain difficult without quantitative survey data.

The high variabilities in coral cover at most sites as shown in the modelled trends and marginal increases in Bayesian *R*^2^ values highlight the importance of separating site-specific and regional trends since they can differ considerably. Indeed, some of the depth variations in the trends uncovered may also be due to differences in the depths surveyed, even though the main trends across depths hold true at the regional level. The rise of other complementary monitoring methods and availability of historical imagery can potentially be utilised in some cases to infill past data by juxtaposing surveyed sites with past images to estimate coral cover for comparisons through time. However, such an approach remains feasible only at much smaller scales and at the most well-documented sites^19^.

Biodiversity is known to be able to buffer against changes in ecosystem state, functioning and resilience^39,58,59^. Thus, the lack of perceived coral cover decline might imply that diversity has been mitigating some of the potential losses. As a consequence of the region’s high diversity of corals and other reef-associated species^35,38^, habitats here may exhibit reduced impacts of stressors and increased resilience^58,59^. Ecological redundancy provided by multiple species performing similar functions on the reef can also confer greater reef resilience^39^. Furthermore, shifts in community composition in response to stressors are possible at the levels of the coral host, algal symbionts and microbiomes^60–63^ for maintaining a high baseline of ecosystem functioning. However, as stressors increase and recovery periods shorten, competitive species with high growth rates may be replaced by more stress-tolerant and weedy species with greater tolerance to environmental variations^64,65^. While competitive species may be able to recolonise reefs in recovery, relentless impacts from current anthropogenic stressors can drive the homogenisation of communities across the components of the holobiont at various geographic scales^66,67^. Aside from coral colony-level changes, reef-scale shifts in community assemblages are not uncommon and have already been reported in multiple studies across the region^68–70^. While some studies have linked these changes to specific causes, such as compositional shifts following bleaching events or cyclone impacts^71–73^, more work needs to be done to identify direct drivers of losses of reef ecosystem resilience over longer timescales.

Despite the lack of benthic cover changes seen in the present study, analyses of the environmental and socio-economic variables suggest the impacts of anthropogenic climate change and coastal development adjacent to reefs in the region are conspicuous, corroborating results of past studies^6,43^. Additionally, while our models do not show any clear effects of bleaching on coral cover, variations observed through the bleaching periods could be indicative of some bleaching impact occurring in tandem with other stressors at various time periods. Higher temperature maxima and variations are associated with bleaching stress, with prolonged bleaching effects potentially leading to coral tissue death and benthic replacement by macroalgae^6,74^. Notably, our analyses have not revealed significant lag effects associated with coral bleaching, suggesting that while bleaching did occur globally, reefs across the large latitudinal range examined here had variable trajectories during and following bleaching events.

While higher net primary productivity can be linked to greater coral and macroalgal photosynthesis and growth, excessive levels and large variations in nutrients can negatively impact coral growth while encouraging macroalgal proliferation^45,75^. The proximity of human populations to reefs has also been hypothesised to adversely affect coral cover, with both larger populations and smaller distances having negative impacts on reef conditions^43,45^ but see also Bruno and Valdivia (2016)^76^. Our results corroborate these general patterns, showing that anthropogenic impacts such as human populations had negative relationships with coral cover alongside positive correlations with macroalgal cover.

The distinct trajectories of East Asian reefs uncovered by this study compared to other monitored regions^30,34,47^ emphasise that regional variations in reef trends must be considered when interpreting findings across spatial scales. Despite encompassing a large reef area, increasing coastal populations and the growing body of research here, the East Asian Seas are relatively understudied and poorly surveyed compared to other regions^11,16,34^. While the results of most reef monitoring studies may be generalisable to global scales and have advanced our understanding of reef dynamics, variations between regions can provide further insights on the interpretation of monitoring data, especially for local reef management. Contextualizing these differences is especially important for making conservation and policy decisions. While coral cover declines in East Asia are not as apparent when compared to other regions, it is important to note that degradation of reef health can manifest as changes in reef community structure and coral species composition instead^64,65,68^. Shifts in community composition can alter the complexity, life history strategies, and functioning of coral reefs^64,65^. For example, phase shifts from coral to algae or other benthic zoantharian or soft coral dominance can have broader implications on reef organisms and the ecosystem services provided by reefs^53,55^. At the local scale, even with few changes in coral assemblage, community functioning can be severely impacted^65,71^. For instance, declines in fast-growing, competitive *Acropora* species with replacement by slower-growing, more stress-tolerant *Porites* species can reduce reef complexity, leading to fewer habitats for reef-associated fish and crabs, and depress carbonate production^64,65,71^. Such changes to reef functioning and the dependent reef fauna can drive further ecosystem declines^39^ and therefore need to be considered in future studies.

This study uncovers the decadal stability of hard coral and macroalgal cover of reefs in the East Asia region, and also underscores the continued need for data and more effective long-term monitoring. The present dataset, while extensive, does not adequately represent the state of most reefs in the region prior to the 1990s. Potentially, some of these data exist but are recorded in the multiple native languages used in East Asia or are stored within governmental organisations and not available for public use^77^. Local research networks are needed to access the data, with inputs to translate and contextualise the associated information, and to provide justifications for use of the data. The collaborative approach of the GCRMN and its members’ networks have been critical for assembling the data presented here, especially from localities that are traditionally underrepresented in coral reef science^78,79^. Expanding these networks would enable greater research participation and make available more relevant data for analysis.

Detailed local knowledge is also necessary for understanding monitoring needs and overcoming data limitations. Long-term monitoring must balance between ease of implementation, availability of resources and trained manpower, as well as data comprehensiveness to identify and substantiate changes through time^80^. Coral and macroalgal cover data on their own are inadequate for precise examination of reef health; more variables are needed to understand how reefs are fundamentally changing^17^. Species-poor stands are not likely to maintain the same level of reef functioning needed for sustained ecosystem health as more diverse reefs^64,81,82^. The high diversity on many East Asian reefs, located within the Coral Triangle and its periphery, stands in contrast against other regions with less speciose reefs^33,36,37^. Recent global biogeographical comparisons have also highlighted the functional redundancies built into the more diverse reefs^40^. While it is difficult to obtain precise species richness estimates throughout all the localities analysed here, many studies of community composition on these reefs do exhibit high diversity^38,45^, which can both confer greater ecosystem redundancy while also increasing the difficulty of taxonomic identification during surveys. The level of reef functioning needs to be quantified and various studies have proposed different suites of variables to be measured and analysed^17,39^.

Here, we suggest a standardised survey methodology, ideally along permanent transects, that at minimum records the genus identities, abundances and sizes of the benthic organisms present, including hard corals (and their growth forms), reef fish and invertebrates^12,80^. Such datasets generate new opportunities to identify major reef changes and evaluate ecosystem functioning, while the methodology remains easy to implement throughout the region. Environmental variables should also be measured in situ alongside these surveys to validate the burgeoning amount of remotely sensed data and enhance the precision of statistical models. Together, these recommendations provide the basis for improved region-wide analyses of reef changes and their timely detection to inform management decisions.

## Methods

### Data processing

Reef surveys were collated from various coordinators of the Global Coral Reef Monitoring Network East Asia (GCRMN EA), including sources from government, non-governmental organisations, research institutes and universities. The ASEAN-Australia Living Coastal Resources project also contributed data for five countries (Indonesia, Malaysia, Philippines, Singapore and Thailand) between 1986 to 1992^83^. Surveys differed in methodology, length and replication, but generally were comparable through transect methodologies for the measurement of benthic cover^84^. Here, to standardise the taxonomic conventions across regions, we define coral or hard coral as Scleractinia and *Heliopora* corals only, while macroalgae is defined as all fleshy seaweed, excluding turf algae. Benthic proportional data were transformed according to Smithson and Verkuilen^85^ to constrain values at the end of the beta scales (0 and 1) and to scale other values in proportion. Missing geographic data were infilled based on other corresponding available information, using either combinations of site names or GPS coordinates or location, and sites were then merged according to relative GPS coordinates. Surveys with missing or unverifiable coral data were removed from the analyses. All analyses were conducted in R^86^ in RStudio^87^. Data processing was performed with ‘dplyr’ v0.8.3, ‘tidyr’ v1.0.2, ‘ggplot2’ v3.2.1 within ‘tidyverse’ v1.3.0.

### Statistics and reproducibility

We used a Bayesian hierarchical model with a beta family distribution implemented in ‘brms’ v2.10.0^88^ to examine patterns in the benthic proportional data and to model coral and macroalgal cover trends through time, with 1 representing 100% benthic cover and 0 representing 0% benthic cover. We used a weakly informative prior based on reef survey values to constrain the models. These priors generally helped to constrain model values to achieve better convergence, but did not strongly change model predictors. Models were first checked for convergence graphically and through model diagnostics using both ‘brms’ diagnostics and ‘performance’ v0.4.4 (Supplementary Table 2, 3). Model parameters were tested and chosen using an information criterion approach with the Widely Applicable Information Criterion (WAIC) and Leave-One-Out Cross Validation (LOO-CV). Random effects, including methods, sites, locations and their combinations were also tested in sequence to identify the best effects for the model. The final hierarchical model utilised a random effect with both spatial variables of site nested within location to better account for geographic variation. Bayesian *R*^2^ values were computed for the final models with both marginal and conditional values to examine variations of fixed and random effects^89^.

### Sensitivity analyses and effects validation

As the dataset was not evenly distributed through time, we divided the dataset for separate analyses to determine if the long-terms trends were masking variations at shorter timescales. Specifically, we subset the dataset into three roughly decadal time periods: 1986–1997 representing the oldest and least data-rich period, 1998–2009 and 2010–2019 with increasing data availability. We also removed two prominent localities independently to see if trends would change due to their disproportionate data coverage and cover—Japan with the richest data and most consistent trends, and Myanmar with the least temporally represented data and disproportionately high coral cover. The reduced datasets were put through the same analysis pipeline as above with the same model structures (Supplementary Fig. 3).

To assess changes in reef cover associated with the major global bleaching events, we extracted three sets of data, each bracketing 2 years prior and following each bleaching event impacting reefs in 1998, 2010 and 2016, to obtain datasets for 1996–2000, 2008–2012 and 2014–2018. We then tested coral cover before, during and after each bleaching event to determine if coral cover changed significantly through these periods using Bayesian hierarchical models incorporating locality, bleaching events, bleaching periods and their interactions, before simplifying the models through ‘brms’ v2.10.0^88^. Model parameters were also tested and chosen using an information criterion approach with WAIC while accounting for the explanatory strength of variables using marginal *R*^2^ resulting in only the bleaching interaction with locality as the random effects. Model verification was done using ‘performance’ v0.4.4. For each site within the full dataset, we identified the year of maximum and minimum coral cover and characterised the change between maximum and minimum cover and its relationship with the time between the two data points.

We also tested for lag effects of the significant temperature variables—maximum sea surface temperature and maximum degree heating weeks. This was done by modelling coral cover against each of the temperature variables separately, with the other variables and year in sequence with year +1, +2 and +3. Model specifications and testing followed the other models above.

### Environmental and socio-economic data

Environmental and socio-economic variables were obtained from Yeager et al.^90^ at 2.5 arcminute intervals to quantify their associations with benthic community trends. These included socio-economic variables such as human population within 5 km and 100 km and distance to nearest market (city centre), and environmental variables such as primary productivity, reef area and wave energy. Human population numbers were converted to annual data approximated from the 5-yearly intervals using both linear (LM) and generalised additive models (GAM) in ‘mgcv’ v1.8–28. GAM approximations were preferred over LM values as they accounted for variable changes in population through time, but LM approximations were used when population values declined substantially by >50%, most probably due to changes in methodology as detailed in Yeager et al.^90^. Temperature data were extracted from the National Oceanic and Atmospheric Administration (NOAA) Coral Reef Watch^91^ at 5 km intervals, from monthly composites of degree heating weeks, sea surface temperatures and anomalies. Means and standard deviations were calculated for these extracted variables. Reef areas within 5 km and 100 km radii were also obtained from Yeager et al.^90^. All sites were then associated with the nearest measurement for that year. The full list of variables can be found in Supplementary Table 6.

We analysed the effects of the above variables on coral and macroalgal cover through time using multi-model averaging. Variables were scaled and centred before selection with the ‘dredge’ function in ‘MuMIn’ v1.43.17, limited to a maximum of 6 variables, following Grueber et al.^92^ using an information theoretic approach. As no distinct top model was identified, conditional model averaging was carried out for all models with ΔAICc values within 6 of the best model^93^.

### Reporting summary

Further information on research design is available in the Nature Portfolio Reporting Summary linked to this article.

## Supplementary information

Supplementary Information

Reporting Summary

## Data Availability

As the data were shared by the various authors under a data sharing agreement, an anonymized dataset with the locations scrubbed is available on the GitHub repository (10.5281/zenodo.7954059). The full dataset can be requested from the corresponding author who will direct the request to the corresponding contributors for approval.

## Code availability

All codes for the analysis have been made available on the GitHub repository (10.5281/zenodo.7954059).
