# Supplementary material for: Decadal stability in coral cover could mask hidden changes on reefs in the East Asian Seas
Source: Commun Biol. 2023 Jun 10;6:630. doi: 10.1038/s42003-023-05000-z (PMC10257672; doi:10.1038/s42003-023-05000-z)
Supplement: Supplementary file 1 — Supplementary Information [file 42003_2023_5000_MOESM1_ESM.pdf]

Supplementary Table 1. Coral reef benthic data sources by locality and respective metadata

Country/State	Survey Method	Length of Surveys (m)	No. of Replicates	Depth Range (m)	Source	Program descriptions and remarks summarized from data providers (Kimura et al. 2022)
Brunei	LIT	30	5-6	4-12	David Lane, Universiti Brunei Darussalam	Brunei reefs were surveyed by David Lane and his team in Universiti Brunei Darussalam using both Reef Check point-intersect transect and line-intersect transect methods from 2005 before Reef Check Brunei took over in 2016. Few sites were surveyed for longer-term periods and historically.
Brunei	Reef Check PIT	20	4	6-18	Reef Check Brunei	
Cambodia	PIT	20	4	2-8	Fauna and Flora International Cambodia	Cambodia reef surveys were led by Fauna and Flora International Cambodia with a modified Reef Check protocol from the 2010s with survey efforts alternating between different archipelagos to build up baseline information of reefs here.
Hong Kong	LIT	30	3	1-3	Reef Check Hong Kong	Reef Check Hong Kong began monitoring reefs in 1997 around Hong Kong, increasing in efforts and the number of sites monitored as the program developed.
Indonesia	PIT, LIT, Photo Transect	50	1-5	3-10	Various NGOs collated through Coral Triangle Center	Indonesia monitoring surveys were conducted through the Coral Reef Rehabilitation and Management Program from the 2000s with most of the data published in the national reports which were not available for the current analysis. Instead, a large number of NGOs and other organisations provided data compiled through the Coral Triangle Center and the Wildlife Conservation Society for a smaller number of selected sites which were then used for this analysis based on similar methodologies and sites which were not being actively restored. While the number of sites here do not accurately reflect most
Indonesia	PIT	50	3	4-11	Wildlife Conservation Society (USAID Sea Project)	

Country/State	Survey Method	Length of Surveys (m)	No. of Replicates	Depth Range (m)	Source	Program descriptions and remarks summarized from data providers (Kimura et al. 2022)
Japan	Timed Swims	50	1	0.5-20	Japan Ministry of the Environment	Japan Ministry of the Environment has led reef monitoring efforts since 1983, with an initial focus on the marine parks around Okinawa prefecture before expanding the survey scope to all other reef areas in 2003. Surveys were conducted using 15-minute timed swims to estimate coral cover only, with no note on macroalgal cover.
Malaysia	Reef Check PIT	20	5	1-16	Reef Check Malaysia	Reef Check Malaysia has been monitoring reefs in Peninsular Malaysia from 2000 and in East Malaysia from 2008, though most of the reef areas are found in East Malaysia around Sabah. Most of the reef sites are also designated through permanent transects.
Myanmar	PIT	20	5	2-4.7	Fauna and Flora Myanmar	Myanmar reef surveys are led by Fauna and Flora International Myanmar, beginning in 2013 using a modified Reef Check protocol across the reef regions though most reef areas. Reef monitoring and studies are relatively new here, and establishment of permanent transects are planned, while most of the previous surveys were aimed at establishing baselines for the reefs here.
Philippines	LIT, Photo Transect	20	1	8.5	Dr Perry Aliño, Community Ecology Lab	Philippine reef surveys have been conducted by various parties, including NGOs, government and academics from the 1990s for the different geographical regions within the Philippines. Much like Indonesia, most of the data available were also not released for the analysis, with certain sites also removed because they were sites for restoration.
Singapore	LIT	20	5	0-10	Reef Ecology Study Team, Reef Ecology Lab	Reef monitoring in Singapore has been undertaken through various research programs from 1986 using line-intersect transects, with different sites surveyed at different frequencies. A long-term monitoring program has continued monitoring for four sites, with other sites being surveyed opportunistically, including on artificial structures which make up much of the modern coastlines.

Country/State	Survey Method	Length of Surveys (m)	No. of Replicates	Depth Range (m)	Source	Program descriptions and remarks summarized from data providers (Kimura et al. 2022)
South Korea	LIT	10	1	5-15	National Marine Ecosystem Survey	Reef surveys in South Korea began relatively recently in 2010 and were conducted during the National Marine Ecosystem Surveys led by the Ministry of Oceans and Fisheries and the Jeju provincial government for three reef sites. Reef areas in South Korea are generally quite limited.
Taiwan	Reef Check PIT	20	4	1.5-14	Taiwan Environmental Information Association	Taiwan reefs have been surveyed using the Reef Check methods since 1997 being undertaken by the Taiwanese Coral Reef Society before the Taiwan Environmental Information Association took over in 2009. These sites encompass most of the reef areas in Taiwan.
Taiwan	Reef Check PIT	50	1	3-18	Taiwanese Coral Reef Society	
Thailand	LIT	15	3-4	0-14	Marine Biodiversity Research Group, Ramkhamhaeng University	Reef monitoring in Thailand have been conducted by the Marine Biodiversity Research Group in Ramkhamhaeng University in collaboration with the Department of Marine and Coastal Resources and the Department of National Parks, Wildlife and Plant Conservation with reef sites across the peninsula, beginning from the 2000s.
Vietnam	Reef Check PIT	20	4	1-14	Institute of Oceanography, Vietnam	In Vietnam, monitoring surveys have been conducted by the team at the Institute of Oceanography, Vietnam Academy of Science and Technology, following the Reef Check protocol across the peninsula at all major reef regions since 2001.
Indonesia, Malaysia, Philippines, Singapore, Thailand	LIT	100	1	0-18.29	ASEAN-Australia Living Coastal Resources	The ASEAN-Australia Living Coastal Resources project was a joint effort between the five ASEAN countries and Australia to pilot long-term monitoring surveys of the regional reefs from 1986 to 1993. Line-intersect transects were used for selected sites across the region to assess an initial baseline of the regional reefs.

Supplementary Table 2. Bayesian Hierarchical Model Coefficients for coral cover

Family: beta; Links: mu = logit; phi = identity

Formula: CoralMin ~ s(Year, by = Locality) + s(Depth) + Locality + (1 | Location/Site)

Data: dataset (Number of observations: 24328); Samples: 4 chains, each with iter = 10000; warmup = 5000; thin = 1; total post-warmup samples = 20000

	Estimate	Est. Error	l-95% CI	u-95% CI	Rhat	Bulk_ESS	Tail_ESS
Smooth Terms:							
sds(sYearLocalityBrunei_1)	0.46	0.33	0.02	1.25	1.00	12436	9161
sds(sYearLocalityCambodia_1)	1.07	0.31	0.55	1.75	1.00	18571	12712
sds(sYearLocalityHongKong_1)	0.87	0.33	0.34	1.6	1.00	16440	12760
sds(sYearLocalityIndonesia_1)	0.47	0.32	0.03	1.21	1.00	10305	7960
sds(sYearLocalityJapan_1)	3.19	0.28	2.66	3.77	1.00	9846	13896
sds(sYearLocalityMalaysia_1)	1.76	0.3	1.23	2.37	1.00	14683	14411
sds(sYearLocalityMyanmar_1)	1.07	0.43	0.22	1.89	1.00	5695	5122
sds(sYearLocalityPhilippines_1)	0.54	0.36	0.03	1.37	1.00	9908	7097
sds(sYearLocalitySingapore_1)	1.12	0.31	0.6	1.8	1.00	10674	10449
sds(sYearLocalitySouthKorea_1)	0.61	0.38	0.04	1.45	1.00	15112	7779
sds(sYearLocalityTaiwan_1)	0.99	0.53	0.06	1.99	1.00	4899	5971
sds(sYearLocalityThailand_1)	1.18	0.37	0.52	1.96	1.00	11094	9436
sds(sYearLocalityVietnam_1)	0.68	0.43	0.06	1.66	1.00	6280	5818
sds(sDepth_1)	2.26	0.29	1.72	2.86	1.00	9341	13172
Group-Level Effects:							
~Location (Number of levels: 219)							
sd(Intercept)	0.43	0.04	0.36	0.51	1.00	3500	6985
~Site (Number of levels: 2031)							
sd(Intercept)	0.65	0.01	0.63	0.68	1.00	3620	7341
Population-Level Effects:							
Intercept	-1.19	0.14	-1.47	-0.91	1.00	4525	7958
LocalityCambodia	0.37	0.31	-0.25	0.99	1.00	7659	11173
LocalityHongKong	-0.06	0.29	-0.62	0.51	1.00	7647	11999
LocalityIndonesia	0.62	0.18	0.27	0.97	1.00	4785	8791
LocalityJapan	-0.11	0.17	-0.43	0.22	1.00	4041	7144
LocalityMalaysia	0.65	0.17	0.32	0.98	1.00	4754	8148
LocalityMyanmar	1.1	0.36	0.4	1.81	1.00	12596	14299
LocalityPhilippines	0.4	0.25	-0.09	0.89	1.00	7904	11592
LocalitySingapore	-0.14	0.21	-0.53	0.27	1.00	5601	10064
LocalitySouthKorea	0.36	0.38	-0.37	1.11	1.00	13528	15125
LocalityTaiwan	0.39	0.22	-0.03	0.82	1.00	5441	9044
LocalityThailand	0.54	0.18	0.19	0.9	1.00	5073	8295
LocalityVietnam	-0.05	0.23	-0.5	0.4	1.00	4568	8349
sYear:LocalityBrunei_1	0.54	0.47	-0.39	1.45	1.00	24819	15331
sYear:LocalityCambodia_1	0.63	0.5	-0.37	1.6	1.00	26921	15221
sYear:LocalityHongKong_1	0.34	0.5	-0.67	1.33	1.00	25282	14861
sYear:LocalityIndonesia_1	0.62	0.46	-0.3	1.5	1.00	17988	15323
sYear:LocalityJapan_1	0.73	0.48	-0.23	1.67	1.00	21641	16043
sYear:LocalityMalaysia_1	0.38	0.49	-0.58	1.34	1.00	26905	14529
sYear:LocalityMyanmar_1	0.4	0.5	-0.58	1.38	1.00	29317	14652
sYear:LocalityPhilippines_1	0.5	0.46	-0.42	1.39	1.00	22212	15636
sYear:LocalitySingapore_1	0.23	0.48	-0.71	1.18	1.00	21956	15388
sYear:LocalitySouthKorea_1	0.49	0.5	-0.5	1.47	1.00	28968	15317
sYear:LocalityTaiwan_1	0.42	0.48	-0.5	1.37	1.00	24479	15112
sYear:LocalityThailand_1	0.6	0.49	-0.37	1.58	1.00	26097	15443
sYear:LocalityVietnam_1	0.71	0.48	-0.27	1.63	1.00	15492	15026
sDepth_1	0.06	0.49	-0.91	1.04	1.00	24113	15477
Family Specific Parameters:							
phi	5.83	0.05	5.72	5.93	1.00	23882	15922

Supplementary Figure 1. Locality-level coral cover smoother across years for Bayesian Hierarchical model

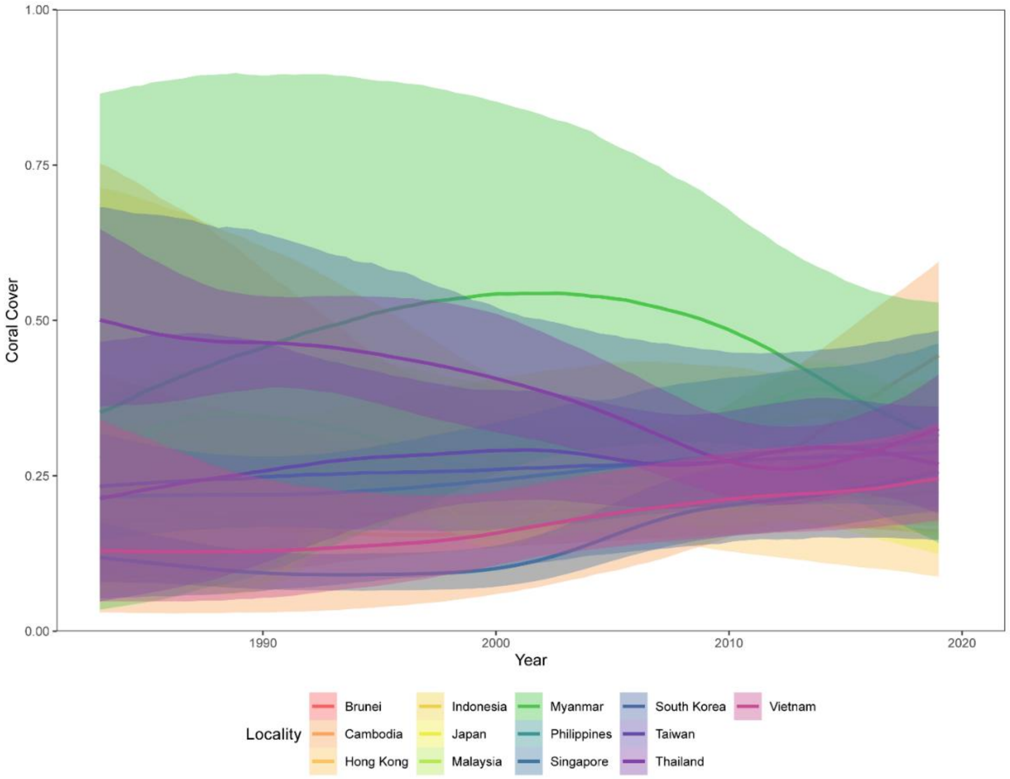

Supplementary Table 3. Bayesian Hierarchical Model Coefficients for macroalgal cover

Family: beta; Links: mu = logit; phi = identity

Formula: AlgaeMin ~ s(Year, by = Locality) + s(Depth) + Locality + (1 | Location/Site)

Data: dataset (Number of observations: 15671); Samples: 4 chains, each with iter = 10000; warmup = 5000; thin = 1; total post-warmup samples = 20000

	Estimate	Est. Error	l-95% CI	u-95% CI	Rhat	Bulk_ESS	Tail_ESS
Smooth Terms:							
sds(sYearLocalityBrunei_1)	0.76	0.16	0.45	1.09	1.00	18977	12738
sds(sYearLocalityCambodia_1)	1.4	0.14	1.14	1.7	1.00	22197	14753
sds(sYearLocalityHongKong_1)	0.2	0.15	0.01	0.57	1.00	16562	8430
sds(sYearLocalityIndonesia_1)	0.63	0.13	0.39	0.91	1.00	17685	13361
sds(sYearLocalityMalaysia_1)	0.2	0.15	0.01	0.56	1.00	16046	8242
sds(sYearLocalityMyanmar_1)	0.2	0.13	0.01	0.53	1.00	9218	6956
sds(sYearLocalityPhilippines_1)	0.2	0.15	0.01	0.55	1.00	14653	8046
sds(sYearLocalitySingapore_1)	0.22	0.15	0.01	0.57	1.00	11615	8690
sds(sYearLocalitySouthKorea_1)	1.09	0.16	0.79	1.42	1.00	17987	14563
sds(sYearLocalityTaiwan_1)	0.2	0.15	0.01	0.57	1.00	15131	7424
sds(sYearLocalityThailand_1)	0.41	0.13	0.19	0.71	1.00	17919	11089
sds(sYearLocalityVietnam_1)	0.52	0.15	0.26	0.84	1.00	14704	8813
sds(sDepth_1)	0.59	0.18	0.23	0.96	1.00	13060	7328
Group-Level Effects:							
~Location (Number of levels: 162)							
sd(Intercept)	0.49	0.05	0.4	0.58	1.00	5137	8343
~Site (Number of levels: 1326)							
sd(Intercept)	0.47	0.02	0.44	0.51	1.00	5317	10436
Population-Level Effects:							
Intercept	-2.86	0.1	-3.06	-2.66	1.00	7364	11549
LocalityCambodia	-0.25	0.21	-0.66	0.17	1.00	16959	15666
LocalityHongKong	-0.33	0.22	-0.76	0.1	1.00	20607	15342
LocalityIndonesia	0.18	0.14	-0.09	0.46	1.00	7008	10686
LocalityMalaysia	0	0.25	-0.48	0.49	1.00	34696	14806
LocalityMyanmar	-0.39	0.12	-0.63	-0.15	1.00	6052	9572
LocalityPhilippines	-0.21	0.23	-0.65	0.23	1.00	17704	14152
LocalitySingapore	0.3	0.18	-0.06	0.64	1.00	12682	14311
LocalitySouthKorea	0.22	0.15	-0.08	0.52	1.00	10693	13766
LocalityTaiwan	0.34	0.23	-0.09	0.79	1.00	22342	15443
LocalityThailand	-0.09	0.17	-0.41	0.24	1.00	9630	11722
LocalityVietnam	0.09	0.18	-0.27	0.44	1.00	11948	14460
sYear:LocalityBrunei_1	0.01	0.18	-0.34	0.36	1.00	7938	11118
sYear:LocalityCambodia_1	0.08	0.25	-0.41	0.57	1.00	32602	15304
sYear:LocalityHongKong_1	0.05	0.25	-0.44	0.52	1.00	33343	14337
sYear:LocalityIndonesia_1	-0.01	0.25	-0.51	0.49	1.00	34901	13785
sYear:LocalityMalaysia_1	-0.12	0.25	-0.61	0.36	1.00	32258	13835
sYear:LocalityMyanmar_1	0	0.25	-0.49	0.49	1.00	34289	14638
sYear:LocalityPhilippines_1	-0.08	0.22	-0.51	0.37	1.00	22823	16003
sYear:LocalitySingapore_1	0	0.25	-0.49	0.49	1.00	34986	14777
sYear:LocalitySouthKorea_1	-0.1	0.24	-0.57	0.38	1.00	28278	14252
sYear:LocalityTaiwan_1	0.02	0.25	-0.46	0.51	1.00	34367	15293
sYear:LocalityThailand_1	0.02	0.25	-0.47	0.51	1.00	35459	14070
sYear:LocalityVietnam_1	0.11	0.25	-0.38	0.6	1.00	31225	14500
sDepth_1	0.1	0.25	-0.39	0.59	1.00	31237	14462
Family Specific Parameters:							
phi	5.23	0.09	5.05	5.41	1.00	21387	15322

Supplementary Figure 2. Locality-level macroalgal cover smoother across years for Bayesian Hierarchical model

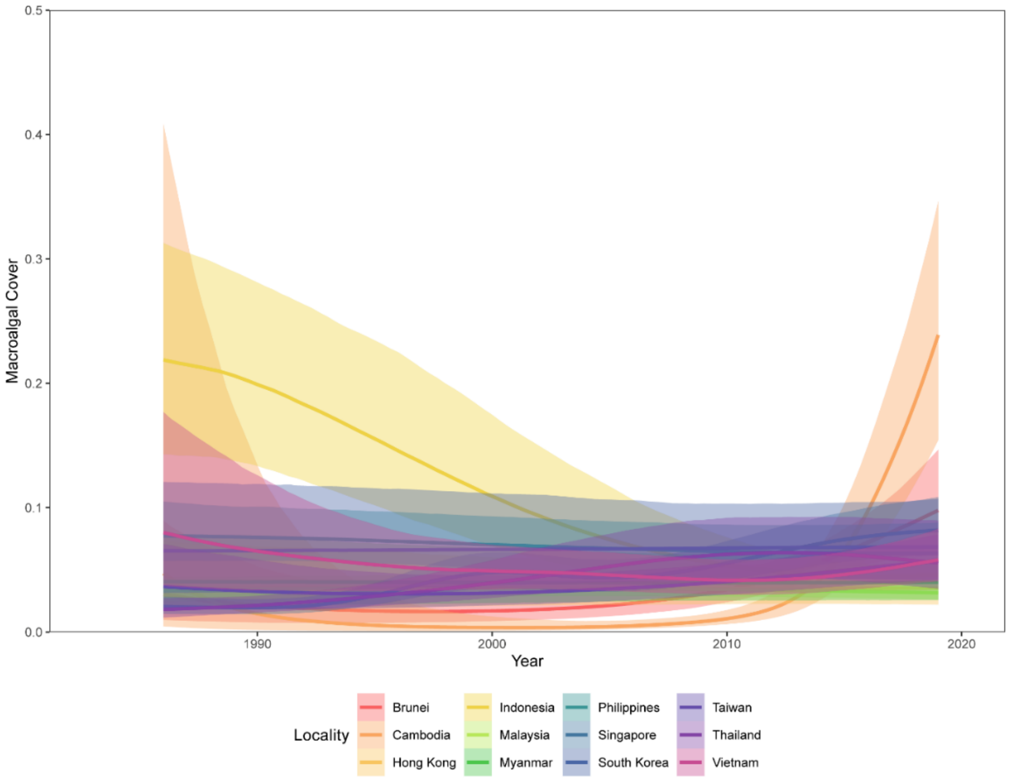

Supplementary Figure 3. Bayesian hierarchical coral cover model plots for datasets after removal of Japan (top) and Myanmar (bottom) respectively showing similar trends with main models

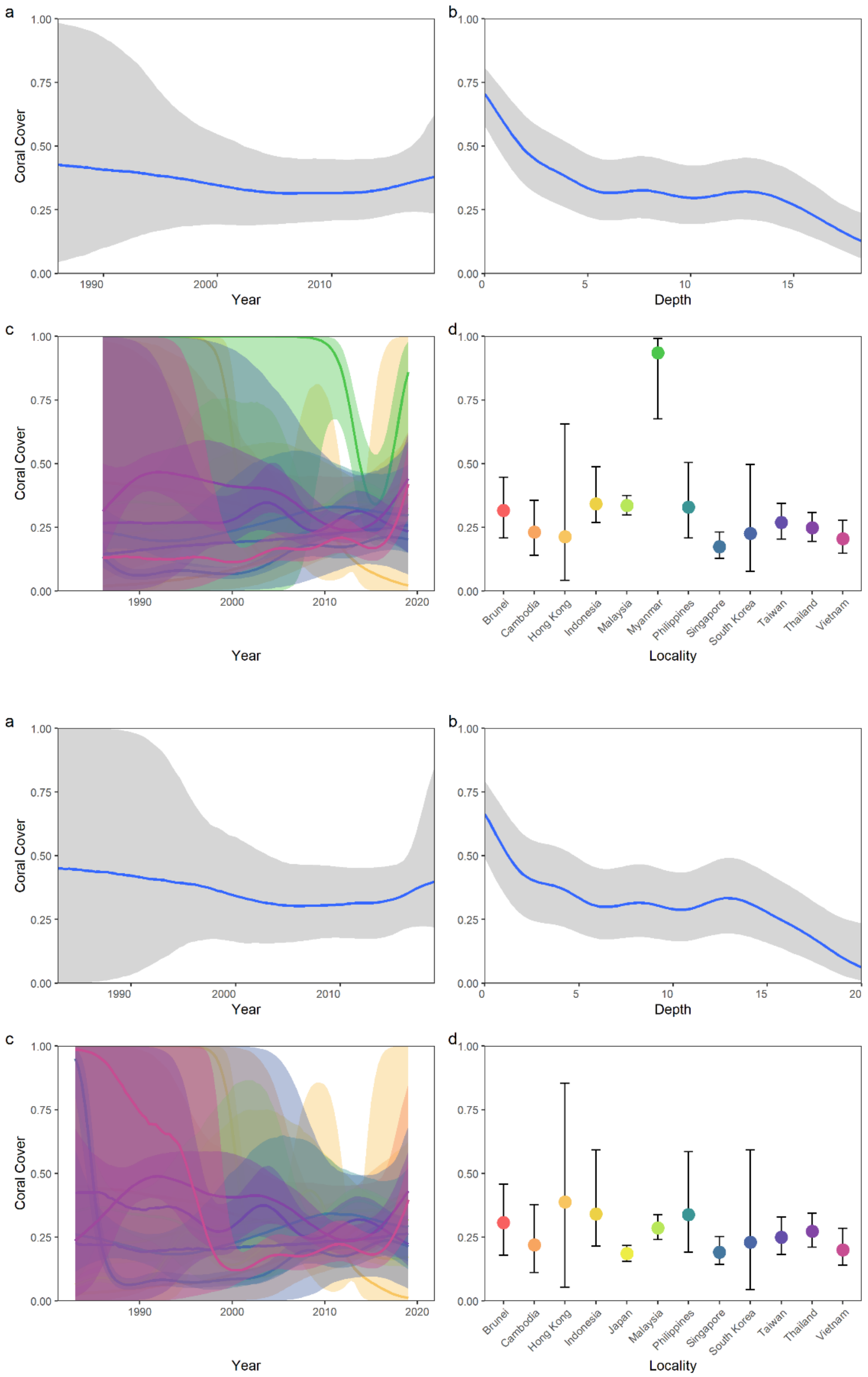

Supplementary Figure 4. Lag effect models for the two temperature-related stress variables maximum Sea Surface Temperatures (SST_max; top) and maximum Degree Heating Weeks (DHW_max; bottom) showing similar patterns across (a) year and (c) year +1 trends alongside temperature trends not changing (b, d)

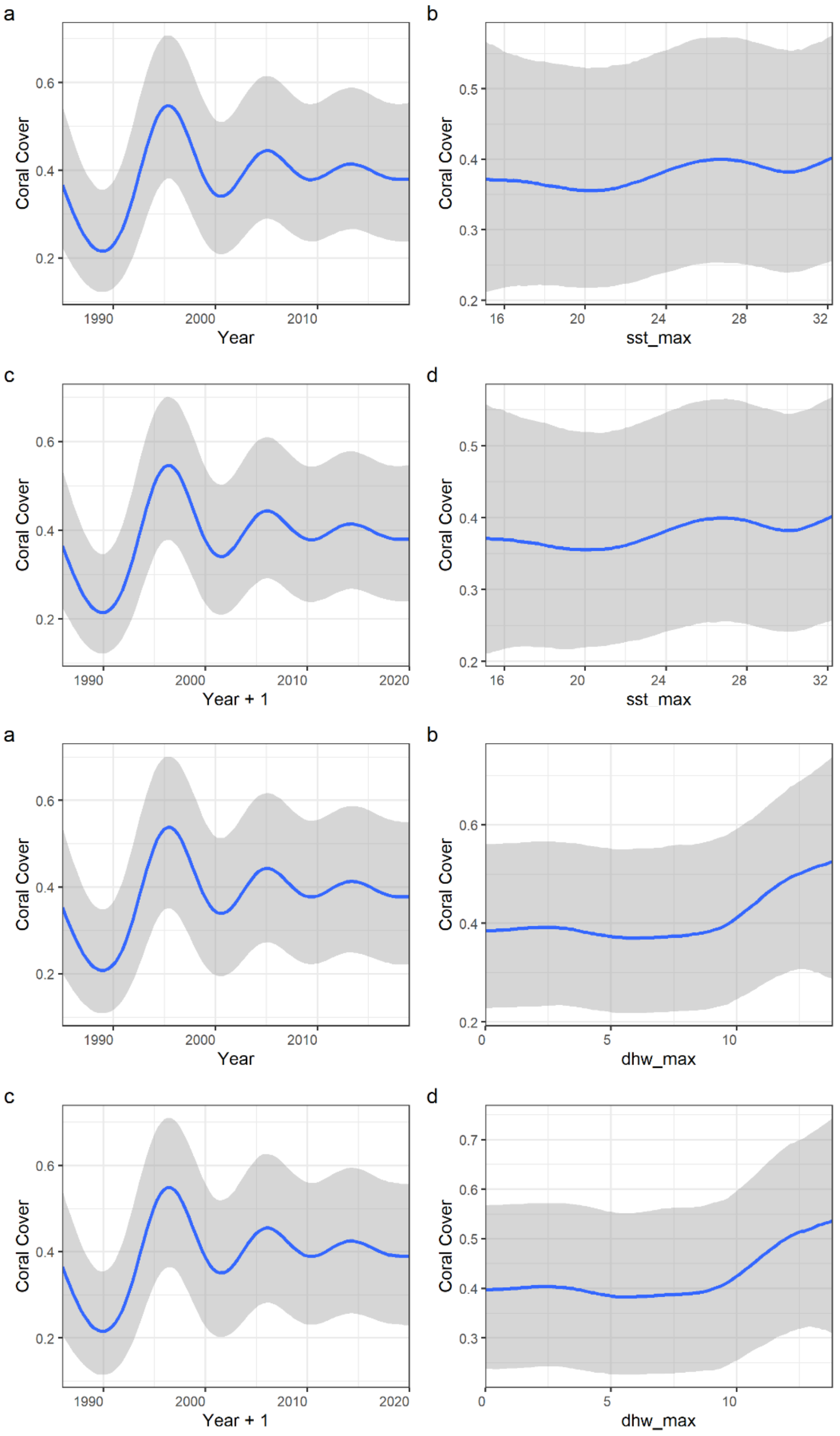

Supplementary Table 4. Full list of multimodal inference models aggregated and variables for coral cover model and their significance values

Components	df	AICc	ΔAICc	Weight
baa_max, locality, depth, npp_max, npp_sd, pop_est_100km	21	-14124.17	0	0.87
locality, depth, npp_max, npp_sd, pop_est_100km, reef_area_100km	21	-14120.35	3.81	0.13

Variable	Effect	Estimate	Adj SE	2.5% CI	97.5% CI	p-value
Intercept		0.3654468	0.0588316	0.25013911	0.480754549	< 2e-16
baa_max	-	-0.0059243	0.0013844	-0.008637659	-0.003210979	1.87e-05
depth	-	-0.0084714	0.0007081	-0.009859313	-0.007083512	< 2e-16
npp_max	+	0.0851812	0.0141532	0.05744144	0.112921	< 2e-16
npp_sd	-	-0.079944	0.0142139	-0.107802632	-0.052085279	< 2e-16
pop_est_100km	-	-0.0392329	0.0063434	-0.051665799	-0.026800096	< 2e-16
reef_area_100km	-	-0.0315939	0.0082055	-0.047676523	-0.015511372	0.000118
Cambodia	N.S.	0.0414715	0.0875706	-0.130163782	0.213106831	0.635801
Hong Kong	+	0.2669813	0.0957564	0.079302182	0.454660338	0.005301
Indonesia	N.S.	0.076208	0.0612552	-0.043850008	0.196265912	0.21346
Japan	N.S.	-0.0551353	0.0624469	-0.177528981	0.067258305	0.377282
Malaysia	N.S.	0.0667011	0.0611692	-0.053188313	0.186590601	0.275521
Myanmar	N.S.	0.1939484	0.1045995	-0.011062817	0.398959546	0.063711
Philippines	N.S.	-0.0830535	0.0708974	-0.222009808	0.055902892	0.241414
Singapore	N.S.	0.1486016	0.1317067	-0.109538803	0.406742096	0.259203
South Korea	N.S.	-0.07925	0.1357178	-0.345252004	0.186751911	0.559266
Taiwan	N.S.	0.0395182	0.070212	-0.098094767	0.17713123	0.573543
Thailand	N.S.	0.0934914	0.0621968	-0.028412171	0.215394988	0.132799
Vietnam	N.S.	-0.0277883	0.0724471	-0.169781932	0.114205362	0.7013

Supplementary Table 5. Full list of multimodal inference models aggregated and variables for macroalgal cover model and their significance values

Components	df	AICc	ΔAICc	Weight
locality, depth, dhw_max, pop_est_100km, sst_max, sst_mean	20	-26778.89	0	0.66
locality, depth, npp_max, pop_est_100km, sst_max, sst_mean	20	-26775.79	3.10	0.14
locality, depth, pop_est_100km, sst_max, sst_mean, year	20	-26774.05	4.84	0.06
locality, depth, dhw_max , pop_est_100km, sst_max, sst_min	20	-26773.92	4.97	0.06
locality, depth, dhw_max, npp_max, pop_est_100km, ssta_max	20	-26773.60	5.29	0.05
locality, depth, npp_sd, pop_est_100km, sst_max, sst_mean	20	-26773.18	5.71	0.04

Variable	Effect	Estimate	Adj SE	2.5% CI	97.5% CI	p-value
Intercept		0.0746795	0.2630684	-0.4409251	0.590284081	0.776503
depth	-	-0.0025499	0.0003963	-0.003326611	-0.00177319	<2e-16
dhw_max	+	0.0028196	0.0009441	0.000969089	0.004670059	0.002823
pop_est_100 km	+	0.0269394	0.0047956	0.01754025	0.036338608	<2e-16
sst_max	-	-0.0158377	0.0036442	-0.02298015	-0.008695182	1.39e-05
sst_mean	+	0.0129316	0.0035174	0.006037559	0.019825567	0.000236
npp_max	+	0.0086851	0.0035592	0.001709261	0.015661005	0.014679
year	+	0.0004912	0.0002452	1.06862E-05	0.000971774	0.045118
sst_min	+	0.0064653	0.0022204	0.002113375	0.010817256	0.003594
ssta_max	-	-0.0041646	0.0009511	-0.006028718	-0.002300497	1.19e-05
npp_sd	+	0.006562	0.0037016	-0.000692922	0.013816964	0.076267
Cambodia	N.S.	-0.0219604	0.0577555	-0.1351591	0.091238275	0.703774
Hong Kong	-	-0.4379972	0.0820659	-0.5988433	-0.277151048	1.00e-07
Indonesia	N.S.	-0.0450449	0.0394184	-0.1223036	0.032213766	0.253147
Malaysia	-	-0.080411	0.0388288	-0.156514	-0.004308031	0.038367
Myanmar	N.S.	-0.1048916	0.0715741	-0.2451743	0.035391167	0.142786
Philippines	N.S.	-0.0161388	0.0427565	-0.09993996	0.067662416	0.705833
Singapore	-	-0.1787021	0.0701397	-0.3161733	-0.041230922	0.01084
South Korea	N.S.	0.0465668	0.0720563	-0.09466094	0.187794565	0.518114
Taiwan	N.S.	-0.0655299	0.0445138	-0.1527755	0.02171558	0.140987
Thailand	N.S.	-0.0622982	0.0405411	-0.1417572	0.017160868	0.124375
Vietnam	N.S.	-0.0394202	0.0466506	-0.1308538	0.052013406	0.398106

Supplementary Table 6. Full list of variables used in multimodal inferences and potential effects on coral/macroalgal cover

Components	Variable	Predicted Effect	Explanation
Year	Year	±	—
Locality	Locality	±	—
Depth	Depth	-	Light attenuates with depth, a similar effect for macroalgae
Estimated population within 5km	pop_est_5km	-	Population density increases anthropogenic pressures
Estimated population within 100km	pop_est_100km	-	Population density increases anthropogenic pressures
Estimated reef area within 5km	reef_area_5km	+	Reef area attenuates localised impacts
Estimated reef area within 100km	reef_area_100km	+	Reef area attenuates localised impacts
Intra-annual Mean NPP	npp_mean	+	NPP increases with increased photosynthesis
Intra-annual Minimum NPP	npp_min	+	NPP increases with increased photosynthesis
Intra-annual Maximum NPP	npp_max	-	Much higher NPP may indicate algae blooms
Intra-annual NPP SD	npp_sd	-	A large variation in NPP may indicate algae blooms
Inter-annual NPP SD	npp_interann_sd	-	A large variation in NPP may indicate algae blooms
Intra-annual mean wave energy	wave_mean	-	High wave energy can damage reefs
Intra-annual wave energy SD	wave_sd	-	High variation in wave energy can damage reefs
Inter-annual wave energy SD	wave_interann_sd	-	High variation in wave energy can damage reefs
Distance to nearest provincial capitals	dist_market	+	Proximity to cities increases anthropogenic impacts
Monthly maximum bleaching alert levels	baa_max	-	Bleaching alert levels highlight potential for bleaching
Monthly maximum Degree Heating Weeks	dhw_max	-	DHWs highlight direct bleaching threat
Monthly maximum SST	sst_max	-	SST increases highlight temperature threats
Monthly mean SST	sst_mean	-	SST increases highlight temperature threats
Monthly minimum SST	sst_min	+	Min SST increases the potential for greater reef growth
Monthly maximum SSTA	ssta_max	-	SSTA (SST anomalies) increases highlight greater temperature variability
Monthly mean SSTA	ssta_mean	-	SSTA increases highlight greater temperature variability
Monthly minimum SSTA	ssta_min	-	SSTA increases highlight greater temperature variability
