# Supplementary material for: Decadal stability in coral cover could mask hidden changes on reefs in the East Asian Seas
Source: Commun Biol. 2023 Jun 10;6:630. doi: 10.1038/s42003-023-05000-z (PMC10257672; doi:10.1038/s42003-023-05000-z)
Supplement: Supplementary file 2 — Reporting Summary [file 42003_2023_5000_MOESM2_ESM.pdf]

nature portfolio

Corresponding author(s): YK Samuel Chan

Last updated by author(s): 09-05-2023

Reporting Summary

Nature Portfolio wishes to improve the reproducibility of the work that we publish. This form provides structure for consistency and transparency in reporting. For further information on Nature Portfolio policies, see our Editorial Policies and the Editorial Policy Checklist.

Statistics

For all statistical analyses, confirm that the following items are present in the figure legend, table legend, main text, or Methods section.

n/a	Confirmed	
☐	☒	The exact sample size (n) for each experimental group/condition, given as a discrete number and unit of measurement
☐	☒	A statement on whether measurements were taken from distinct samples or whether the same sample was measured repeatedly
☐	☒	The statistical test(s) used AND whether they are one- or two-sided<br>*Only common tests should be described solely by name; describe more complex techniques in the Methods section.*
☐	☒	A description of all covariates tested
☐	☒	A description of any assumptions or corrections, such as tests of normality and adjustment for multiple comparisons
☐	☒	A full description of the statistical parameters including central tendency (e.g. means) or other basic estimates (e.g. regression coefficient) AND variation (e.g. standard deviation) or associated estimates of uncertainty (e.g. confidence intervals)
☐	☒	For null hypothesis testing, the test statistic (e.g. F, t, r) with confidence intervals, effect sizes, degrees of freedom and P value noted<br>*Give P values as exact values whenever suitable.*
☐	☒	For Bayesian analysis, information on the choice of priors and Markov chain Monte Carlo settings
☐	☒	For hierarchical and complex designs, identification of the appropriate level for tests and full reporting of outcomes
☐	☒	Estimates of effect sizes (e.g. Cohen's d, Pearson's r), indicating how they were calculated

Our web collection on statistics for biologists contains articles on many of the points above.

Software and code

Policy information about availability of computer code

Data collection	Data files were aggregated from various data providers through a data sharing agreement, manually checked for discrepancies, and then aggregated in R for analysis.
Data analysis	Bayesian Hierarchical Generalised Additive Models were used for most analyses to derive coral and macroalgal trends across years. Multimodal inferences were used to determine correlations between socio-economic and environmental variables with coral and macroalgal cover.

For manuscripts utilizing custom algorithms or software that are central to the research but not yet described in published literature, software must be made available to editors and reviewers. We strongly encourage code deposition in a community repository (e.g. GitHub). See the Nature Portfolio guidelines for submitting code & software for further information.

Data

Policy information about availability of data

All manuscripts must include a data availability statement. This statement should provide the following information, where applicable:

- Accession codes, unique identifiers, or web links for publicly available datasets
- A description of any restrictions on data availability
- For clinical datasets or third party data, please ensure that the statement adheres to our policy

Data in this article was made available via a data sharing agreement for the purposes of this study, as not all data are publicly available. These can be made available upon request through the corresponding author and the various data providers.

Human research participants

Policy information about studies involving human research participants and Sex and Gender in Research.

Reporting on sex and gender	NA
Population characteristics	NA
Recruitment	NA
Ethics oversight	NA

Note that full information on the approval of the study protocol must also be provided in the manuscript.

Field-specific reporting

Please select the one below that is the best fit for your research. If you are not sure, read the appropriate sections before making your selection.

☐ Life sciences ☐ Behavioural & social sciences ☒ Ecological, evolutionary & environmental sciences

For a reference copy of the document with all sections, see nature.com/documents/nr-reporting-summary-flat.pdf

Ecological, evolutionary & environmental sciences study design

All studies must disclose on these points even when the disclosure is negative.

Study description	The study aims to identify trends for coral and macroalgal cover through time (year) and across regions (localities), using a Bayesian hierarchical Generalised Additive Model with sites and locations as nested random effects. Smoothers were used to generate non-linear trends to understand major reef benthic trends. Socio-economic and environmental correlates were modelled through a multimodal inference averaging approach against coral and macroalgal cover to understand which variables were associated with high and low cover.
Research sample	Reef survey data were provided by the various data providers from different regions.
Sampling strategy	All data from providers were checked for completeness before being used in the models.
Data collection	Reef survey data were collated from various data providers based on available data. Surveys were collected by various organisations. Environmental and socio-economic correlates were extracted from online databases using GPS locations.
Timing and spatial scale	Surveys collated ranged from 1983 to 2019 across the East Asian reef region.
Data exclusions	All data from providers which were complete were used.
Reproducibility	A start seed was used to ensure analyses were reproducible. Codes are made available on github.
Randomization	No randomization was conducted. All data that were made available were used whenever possible.
Blinding	No blinding was used as survey data had to be labelled and location-specific.

Did the study involve field work? ☐ Yes ☒ No

Reporting for specific materials, systems and methods

We require information from authors about some types of materials, experimental systems and methods used in many studies. Here, indicate whether each material, system or method listed is relevant to your study. If you are not sure if a list item applies to your research, read the appropriate section before selecting a response.

Materials & experimental systems

n/a	Involved in the study	
☒	☐	Antibodies
☒	☐	Eukaryotic cell lines
☒	☐	Palaeontology and archaeology
☒	☐	Animals and other organisms
☒	☐	Clinical data
☒	☐	Dual use research of concern

Methods

n/a	Involved in the study	
☒	☐	ChIP-seq
☒	☐	Flow cytometry
☒	☐	MRI-based neuroimaging
